# Genetic variation for root architectural traits in response to phosphorus deficiency in mungbean at the seedling stage

**DOI:** 10.1371/journal.pone.0221008

**Published:** 2020-06-11

**Authors:** Venkata Ravi Prakash Reddy, Muraleedhar S. Aski, Gyan Prakash Mishra, Harsh Kumar Dikshit, Akanksha Singh, Renu Pandey, Madan Pal Singh, Gayacharan, Vinita Ramtekey, Priti, Neha Rai, Ramakrishnan M. Nair

**Affiliations:** 1 Division of Genetics, ICAR-Indian Agricultural Research Institute, New Delhi, India; 2 Amity Institute of Organic Agriculture, Amity University, Noida, India; 3 Division of Plant Physiology, ICAR-Indian Agricultural Research Institute, New Delhi, India; 4 Germplasm Evaluation Division, ICAR-National Bureau of Plant Genetic Resources, New Delhi, India; 5 World Vegetable Center, South Asia/Central Asia, Patancheru, Hyderabad, India; Wageningen University, NETHERLANDS

## Abstract

Roots enable the plant to survive in the natural environment by providing anchorage and acquisition of water and nutrients. In this study, root architectural traits of 153 mungbean genotypes were compared under optimum and low phosphorus (P) conditions. Significant variations and medium to high heritability were observed for the root traits. Total root length was positively and significantly correlated with total root surface area, total root volume, total root tips and root forks under both optimum P (r = 0.95, r = 0.85, r = 0.68 and r = 0.82 respectively) and low P (r = 0.95, r = 0.82, r = 0.71 and r = 0.81 respectively). The magnitudes of the coefficient of variations were relatively higher for root forks, total root tips and total root volume. Total root length, total root surface area and total root volume were major contributors of variation and can be utilized for screening of P efficiency at the seedling stage. Released Indian mungbean varieties were found to be superior for root traits than other genotypic groups. Based on comprehensive P efficiency measurement, IPM-288, TM 96–25, TM 96–2, M 1477, PUSA 1342 were found to be the best highly efficient genotypes, whereas M 1131, PS-16, Pusa Vishal, M 831, IC 325828 were highly inefficient. Highly efficient genotypes identified would be valuable genetic resources for P efficiency for utilizing in the mungbean breeding programme.

## Introduction

Mungbean is an important warm season grain legume grown in more than 6 million hectares area [1] for its protein rich seeds. Mungbean seeds are rich source of iron [2], vitamin C and folates [3]. Cultivation of mungbean improves soil fertility through biological nitrogen fixation [4]. Mungbean is cultivated on marginal lands resulting in poor growth, development and yield. Therefore, fertilizer management is important for realizing the potential yield of the crop [5]. Nitrogen (N) and phosphorus (P) are the important macronutrients required for the crop. In mungbean, 80–90% N requirement is met through biological N_2_ fixation [6] however, it requires 48.1 kg P_2_O_5_ for producing one ton of grains [7]. Under tropical and subtropical conditions, P is the main yield limiting factor [8].

Globally, by the year 2020, P fertilizer requirement is expected to reach 64.68 million tonnes, whereas, the estimated supply is 53.08 million tonnes and the demand for P fertilizer requirement is increasing annually by 2.2% on an average from 2015–2020 [9]. Countries like US, China and Morocco are the leading producers of phosphatic fertilizer [10]. In anticipation of future domestic demands, the US and China have stopped the export of rock phosphate to other countries [11]. The deficiency of P leads to a higher root/shoot ratio as shoot growth is relatively more affected in comparison to root growth [12]. It also causes stunted growth and foliage turns dark green color with reddish-purple tips and leaf margins due to the accumulation of starch and anthocyanin in the leaves [13]. Deficiency in leaves disturbs the photosynthetic machinery and electron transport chain through repression of orthophosphate concentration in chloroplast stroma inhibiting ATP synthase activity [14]. P is a key component of nucleic acids, membrane lipids and participates in energy transfer reactions, and thus determines the yield and quality of a crop [15, 16]. Its deficiency in soil can be overcome by P fertilizer application, but excess application leads to the delayed formation of reproductive organs [17]. Acquisition of P from the soil is a complex process as it is bound to calcium in alkaline soils and iron and aluminium in acid soils [18].

Change in root architecture explores the soil space and thus enhanced root-soil contact increases P efficiency [19–21]. The rate of nutrient acquisition by plant roots depends upon the particular nutrient concentration at the root surface, root properties and plant requirements [22]. The root is an indispensable organ of the plant for the absorption of nutrients and water by expanding its surface area and enhancement of explored soil volume [23]. Under low P conditions, plants modify their root architectural traits [23, 24] which include reduced primary root growth, increase in number and length of lateral roots and root hairs [25–27], increase in root surface area and volume [28], shallower root growth angle [29] and enhancement of root biomass [30] for enhancement of phosphorus uptake.

Genetic variation in plant root architecture can be exploited to improve the nutrient and water use efficiency under difficult growing conditions [31]. Root surface area, volume, biomass and root carboxylate exudation capacity were reported to be significantly higher in P efficient mungbean genotype compared to inefficient genotype [32]. The significant contribution of root length, root volume, surface area and the number of lateral roots towards P uptake at 45 days after sowing was observed in blackgram [28]. P deficiency causes a significant increase in primary root length, total root length and number of lateral roots after eight days of treatment in lentil [33]. High adventitious and lateral root densities were associated with high P uptake per unit length in soybean and common bean [34, 35]. Genotypes with a large root system with deep lateral roots exhibited high shoot and root P use efficiency compared to genotypes with medium and small root system in lupin [36]. In rice, root hair length and density significantly increased in all tested genotypes under low P conditions [37]. Shen *et al*. [38] stressed on maintaining root biomass and root length to cope with the deficiency of P in wheat. Under low P condition, decrease in root length was more in fibrous root species (wheat, rapeseed than in legumes (broad bean, soybean, chickpea and lupin). Maize and wheat had a higher root to shoot ratio and rapeseed had higher specific root length than legumes [39].

Although P deficiency can affect crop growth throughout the season, phenotypic evaluation at the seedling stage is an attractive approach, as it is high throughput and low-cost, which saves time and space [40]. Stress gradient hypothesis [41, 42] proposes that the fate of seedlings determines the structure and dynamics of the plant population. Current digital image analysis enables accurate analysis of plant root system and is time and labour-saving technology [43, 44]. Considering the role of root architecture in P efficiency, the present study was designed to (i) characterize the phenotypic variation for morphological root traits in 153 mungbean genotypes, (ii) identify the root related traits accounting for most of the variation among the tested mungbean genotypes, and (iii) evaluate the efficiency of mungbean genotypes under optimum and low P conditions.

## Materials and methods

### Plant materials and plant growth conditions

One hundred and fifty-three mungbean genotypes including 41 Indian released varieties (IRV), 44 Advanced Breeding lines (ABL)and 68 Germplasm lines (GL) were studied for root architecture characteristics under optimum P (OP) and low P(LP) conditions (S1 Table). The experiment was conducted in a National Initiative on Climate Resilient Agriculture (NICRA)-controlled environment facility of the Indian Agricultural Research Institute, New Delhi, India from December 2017 to September 2018. The growth conditions in the greenhouse were maintained as 30/18°C day/night temperature, photoperiod of 12 h and relative humidity at 90%. For screening under hydroponics, mungbean seeds were surface sterilized with 0.1% (w/v) HgCl_2_ for 3 minutes followed by rinsing with double distilled water and wrapped in germination paper. Upon the emergence of cotyledonary leaves, seedlings of uniform size and without visible root injuries were transferred to the modified Hoagland solution. Composition of basal nutrient solution used was MgSO_4_ (1mM), K_2_SO_4_(0.92 mM), CaCl_2_.2H_2_O (0.75 mM), Fe-EDTA (0.04mM), Urea (5 mM), and micronutrients [H_3_BO_3_(2.4μM), MnSO_4_ (0.9μM), ZnSO_4_ (0.6μM), CuSO_4_ (0.62μM), and Na_2_MoO_4_ (0.6μM)] [45]. Two levels of P were maintained using KH_2_PO_4_ as optimum P (250 μM) and low P (3μM). A preliminary experiment was conducted with a series of P concentrations to select the optimum and low P levels. Analysis of observations on biomass, chlorophyll content and visual symptoms led to the selection of optimum (250μM) and low (3 μM) P concentration (data not presented) (S1 Fig). The chlorophyll concentration was measured by using MC-100 chlorophyll concentration meter (Apogee Instrumnets, Inc., USA). The biomass was calculated by drying the plants at 60°C until obtaining the constant mass of dry weight. The pH of the nutrient solution was maintained at 6.0 using 1 M KOH or 1 M HCL. Seedlings were supported on a 2” thick thermocol sheet with holes made at 5 × 5 cm plant-to-plant and row-to-row distance. This sheet was fitted into plastic containers (30 × 45 × 15 cm) with 10 L of basal nutrient solution. Forty-five seedlings were raised in one such container and fifteen genotypes were screened at a time with three replicates for each genotype. The solution was aerated regularly by aquarium air pump and replaced on alternate days.

### Root measurements

The data on root traits were recorded on twenty-one days old seedlings raised under low and optimum P conditions. The complete root system was isolated from each plant and spread out in a tray with no overlapping of roots. Roots were scanned using root scanner (Epson professional scanner) and greyscale images obtained in TIFF format were analyzed using WinRhizo (Pro version 2016a; Regent Instrument Inc., Quebec, Canada). The settings were used as follows: image resolution, 400 dpi; calibration, intrinsic for the scanner; manual—dark root on white background; bit depth (8 –bit); focal length (0 mm); image dimensions (4395 × 6125 pixels). Roots were placed in a 30 × 40 × 2 cm size acrylic tray with 700 ml water. During root scanning, the debris consisting of occasional broken root segments were manually separated from the root sample by floating them in trays containing water. The recovered clean roots were used for scanning. The following root parameters were obtained: primary root length (PRL), total root length (TRL), total root surface area (TSA), total root volume (TRV), root average diameter (RAD), total root tips (TRT), and root forks (RF). PRL was measured manually using the scale. TRL represents the sum of primary, seminal, crown, basal and lateral roots.

WinRhizo also generated additional output that allowed us to categorize root traits root length (RL), root surface area (RSA), root volume (RV) and number of root tips (RT) into five classes based on root diameter intervals of 0–0.5 mm, 0.5–1.0 mm, 1.0–1.5 mm, 1.5–2.0 mm and >2.0 mm [46–48].

### Statistical analysis

The P efficiency coefficient (PEC) was calculated as the ratio of the data derived from the low P (LP) and optimum P (OP) treatment of the same genotype for each trait using the following equation.

$$PEC_{ij}=X_{ijLP}/X_{ijOP}$$

Where *PEC*_*ij*_ is the P efficiency coefficient of the trait (*j*) for the cultivar (*i*); *X*_*ijLP*_ and *X*_*ijOP*_ are the value of the root trait (*j*) for the cultivar (*i*) evaluated under low P (LP) and optimum P (OP) treatments, respectively.

The data were subjected to descriptive statistics including mean, standard deviation, coefficient of variation, analysis of variation, heritability and Pearson’s correlation were calculated for tested traits under OP and LP conditions using STAR (Statistical Tool for Agricultural Research) 2.1.0 software [49]. For performing analysis of variance of root traits, the model used was a fixed factor model with both genotype and P as fixed factors. The additive linear model used was:

Yijk=μ+Gi+Pi+(GP)ij+Eijk

where *Yijk* is the observation from *k*^*th*^ replicate of *ij*^*th*^ experimental unit, *μ* is the overall mean, *Gi* is the main effect of *i*^*th*^ genotype, *Pj* is the main effect of *j*^*th*^ P level, *GP*^*ij*^ is the interaction effect between genotype and P, *Eijk* is the random effect error confounded in the experiment.

The proportion of root traits in each diameter class was calculated as the percentage of the total trait under OP and LP conditions in different groups [48]. Mungbean genotypes (153) were classified into three different categories based on their performance: (i) low performing genotypes ($\leq \bar{\text{x}}-\text{SD}$), (ii) medium performing genotypes ($\geq \bar{\text{x}}-\text{SD}$) to $\leq \bar{\text{x}}+\text{SD}$), and (iii) high performing genotypes ($\geq \bar{\text{x}}+\text{SD}$), where $\bar{\text{x}}$ and SD are mean and standard deviation of respected root trait [50, 51]. A polymorphic diversity index, Shannon-Weaver diversity index (H’), was calculated for each trait [52–54] using the formula:

$$\text{H}’=-\sum_{i=1}^{s}pi(\text{ln}pi)$$

Where *pi* is the proportion of individuals belonging to the i^th^ class and s is the total number of genotypes.

The principal component analysis was performed to identify traits contributing most of the variation in tested mungbean genotypes using STAR 2.1.0 software. A comprehensive P efficiency measurement value (CPEM value) was used to estimate the efficiency capability of all tested mungbean genotypes. The CPEM value was calculated across traits to evaluate mungbean P efficiency by using the formulas described below [46, 55].

Fuzzy subordination method could be used to analyze the P efficiency completely and avoid the shortage of single index. The membership function of a fuzzy set is a generalization of the indicator function in classical sets; it represents the degree of truth as an extension of valuation [46, 56 and 57]. *U*_*ij*_ stands for the membership function value of P efficiency (MFVP) that indicates a positive correlation between the trait and P efficiency.

$$Uij=\frac{PECij-PECjmin}{PECjmax-PECjmin}(\text{j}=1,2,3\ldots \text{n})$$

Where *U*_*ij*_ is the membership function value of the trait (*j*) for the cultivar (*i*) for P efficiency; *PEC*_*jmax*_ is the maximum value of the P efficiency coefficient for the trait (*j*); *PEC*_*jmin*_ is the minimum value of *PEC*_*j*_.

Comprehensive P efficiency measurement was made using the formula:

$$\text{P}=\sum_{j=1}^{n}[Uij\times \left|PECij\right|/\sum_{j=1}^{n}\left|PECij\right|]\;(\text{j}=1,2,3\ldots \text{n})$$

Where CPEM is the comprehensive P efficiency measurement of each mungbean genotype under LP condition. Based CPEM value all mungbean genotypes were classified into five groups, highly efficient, efficient, moderately efficient, inefficient and highly inefficient.

## Results

### Response of root traits to phosphorus stress

The study of 153 mungbean genotypes for root traits under optimum and low P conditions revealed a high variation in the mean values for the studied traits (Table 1). Independent *t*-test at the level of significance 0.05 indicated that the mean values of PRL (p-value 0.04) and RAD (p-value <0.01) were significantly high in LP as compared to OP condition. For P efficiency coefficient (PEC), RAD showed high mean value followed by TRV and PRL. The mean values of RL, RSA, RV and RT in five root diameter classes: 0–0.5 mm, 0.5–1.0 mm, 1.0–1.5 mm, 1.5–2.0 mm and >2.0 mm exhibited variation under OP and LP conditions. RL and RSA revealed PEC value above 1 for all root diameters except 0–0.5 mm. The PEC was above 1.0 for RV at all root diameters indicating an increase in root volume in LP condition. The RT was higher under LP in the root diameter class of 0.5–1.0 mm.

**Table 1:** Mean value, Standard Deviation (SD) of traits investigated under two phosphorus regimes and the Phosphorus Efficiency-Coefficient (PEC) of each trait.

Trait	Mean ± SD (LP)	Mean ± SD (OP)	Mean ± SD (PEC)
PRL	36.04 ± 6.38	34.78 ± 6.40	1.06 ± 0.22
TRL	809.34 ± 180.07	897.67 ± 209.74	0.92 ± 0.17
TSA	85.58 ± 19.32	89.48 ± 22.54	0.98 ± 0.19
TRV	0.74 ± 0.19	0.71 ± 0.21	1.07 ± 0.24
RAD	0.34 ± 0.02	0.31 ± 0.02	1.08 ± 0.06
TRT	788.62 ± 248.04	964.77 ± 291.77	0.85 ± 0.26
RF	1817.32 ± 583.50	2495.68 ± 735.22	0.74 ± 0.20
RL^1^	719.50 ± 158.99	815.93 ± 175.23	0.89 ± 0.17
RL^2^	64.31 ± 23.85	59.96 ± 24.05	1.12 ± 0.29
RL^3^	6.31 ± 1.74	6.23 ± 1.96	1.06 ± 0.29
RL^4^	2.51 ± 0.78	2.42 ± 0.87	1.14 ± 0.48
RL^5^	0.35 ± 0.18	0.41 ± 0.21	1.08 ± 1.48
RSA^1^	57.46 ± 12.14	59.90 ± 13.63	0.98 ± 0.19
RSA^2^	12.68 ± 4.58	11.86 ± 4.63	1.11 ± 0.29
RSA^3^	2.37 ± 0.65	2.34 ± 0.73	1.06 ± 0.29
RSA^4^	1.35 ± 0.42	1.30 ± 0.47	1.14 ± 0.48
RSA^5^	0.29 ± 0.17	0.36 ± 0.19	1.11 ± 1.77
RV^1^	0.41 ± 0.09	0.40 ± 0.10	1.05 ± 0.22
RV^2^	0.21 ± 0.07	0.19 ± 0.07	1.11 ± 0.29
RV^3^	0.07 ± 0.02	0.07 ± 0.02	1.07 ± 0.29
RV^4^	0.06 ± 0.02	0.06 ± 0.02	1.14 ± 0.48
RV^5^	0.02 ± 0.01	0.03 ± 0.02	1.20 ± 2.26
RT^1^	772.15 ± 232.56	951.37 ± 277.88	0.84 ± 0.25
RT^2^	6.64 ± 2.70	5.28 ± 2.20	1.48 ± 0.98
RT^3^	0.49 ± 0.49	0.46 ± 0.50	0.89 ± 1.17
RT^4^	0.27 ± 0.35	0.18 ± 0.30	0.39 ± 0.52
RT^5^	0.05 ± 0.05	0.06 ± 0.06	0.37 ± 0.49

Independent *t*-test indicated that the mean values of PRL and RAD were significantly high in LP condition compared to OP condition. LP, low phosphorus; OP, optimum phosphorus; PRL, primary root length; TRL, total root length; TSA, total root surface area; TRV, total root volume; TRT, total root tips; RAD, root average diameter; RF, root forks; RL^1-5^, RSA^1-5^, RV^1-5^, RT^1-5^ indicate average root length, root surface area, root volume and root tips in diameter between 0.0 and 0.5 mm, 0.5 and 1.0 mm, 1.0 and 1.5 mm, 1.5 and 2 mm and greater than 2.0 mm respectively.

### Genetic variation and broad sense heritability studies

**Fig 1:**
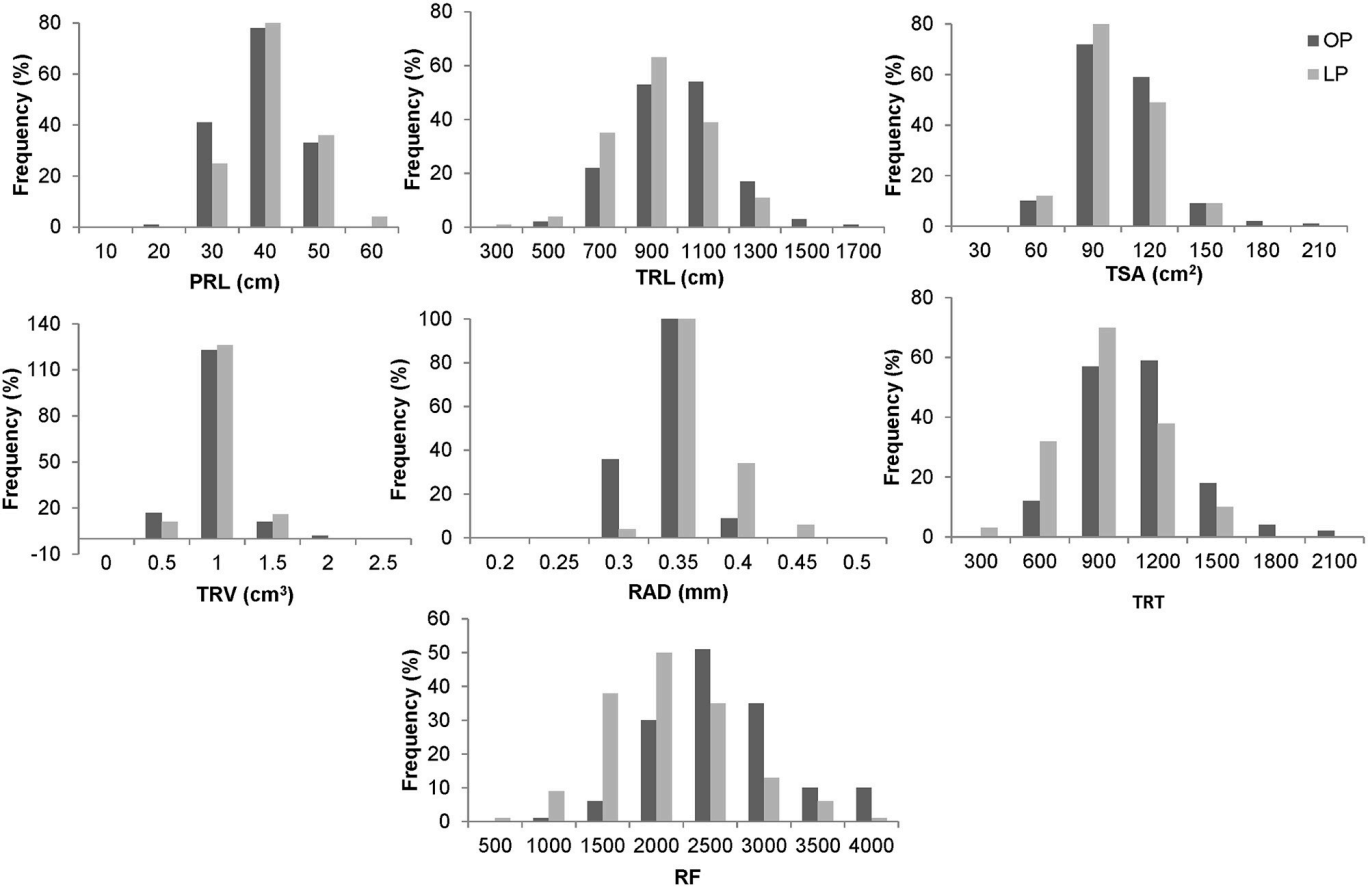
Frequency distribution of variation for seven root traits in 153 mungbean lines. PRL, primary root length; TRL, total root length; TSA, total root surface area; RAD, root average diameter; TRV, total root volume; TRT, total root tips; RF, root forks; OP, optimum phosphorus condition; LP, low phosphorus condition.

ANOVA analysis revealed highly significant variation among the genotypes for seven traits (PRL, TRL, TSA, TRV, RAD, TRT and RF) evaluated under two P regimes (Table 2). The study revealed highly significant variation among the evaluated traits at two P conditions. The highly significant interaction between genotype and P treatment indicates that genotypes were significantly affected for studied root traits at different P regimes. The level of variation for studied seven P efficiency traits is presented as Fig 1. Histogram of frequency distribution revealed near normal distribution of root traits evaluated in the study. The coefficient of variation for seven investigated traits ranged from 4.64% (RAD) to 16.01% (RF). The broad sense heritability for the studied traits ranged from 0.59 to 0.79. The highest broad sense heritability was observed in RAD (0.79) followed by TRV (0.78) and lowest was observed in PRL (0.59).

**Table 2:** Analysis of variance for the tested traits under two phosphorus regimes.

Variables	Mean squares				CV (%)	Heritability
	Replication (R)	Genotype (G)	Phosphorus (P)	G × P		
df	2	152	1	152		
PRL	39.48 (0.032)	174.33 (<0.001)	376.63 (<0.001)	70.89 (<0.001)	9.55	0.59
TRL	83366.00 (<0.001)	183719.63 (<0.001)	1789765.97 (<0.001)	45566.49 (<0.001)	11.96	0.75
TSA	874.45 (<0.001)	2151.76 (<0.001)	3503.19 (<0.001)	491.44 (<0.001)	12.40	0.77
TRV	0.08 (0.001)	0.20 (<0.001)	0.18 (0.001)	0.04 (<0.001)	15.26	0.78
RAD	0 (0.93)	0.00 (<0.001)	0.14 (<0.001)	0.00 (<0.001)	4.64	0.79
TRT	21779.66 (0.311)	321101.87 (<0.001)	7121141.19 (<0.001)	118860.09 (<0.001)	15.58	0.63
RF	364416.28 (0.047)	2094494.45 (<0.001)	105608699.30 (<0.001)	548698.57 (<0.001)	16.01	0.74
RL^1^	65020.02 (0.017)	138491.6 (<0.001)	1796062.03 (<0.001)	43826.93 (<0.001)	16.11	0.68
RL^2^	628.35 (0.021)	3405.73 (<0.001)	4450.00 (<0.001)	634.44 (<0.001)	20.32	0.81
RL^3^	0.98 (0.577)	15.63 (<0.001)	0.55 (0.58)	5.87 (<0.001)	21.35	0.62
RL^4^	0.93 (0.236)	2.80 (<0.001)	2.97 (0.031)	1.17 (<0.001)	33.03	0.58
RL^5^	0.01 (0.697)	0.17 (<0.001)	1.20 (<0.001)	0.09 (<0.001)	38.06	0.47
RSA^1^	264.11 (0.076)	813.99 (<0.001)	2472.15 (<0.001)	274.26 (<0.001)	17.08	0.66
RSA^2^	20.82 (0.034)	133.11 (<0.001)	148.84 (<0.001)	22.01 (<0.001)	19.90	0.83
RSA^3^	0.23 (0.546)	2.15 (<0.001)	0.56 (0.223)	0.78 (<0.001)	25.95	0.64
RSA^4^	0.31 (0.227)	0.82 (<0.001)	0.12 (0.441)	0.35 (<0.001)	33.76	0.57
RSA^5^	0.00 (0.994)	0.15 (<0.001)	1.47 (<0.001)	0.08 (<0.001)	50.23	0.47
RV^1^	0.02 (0.038)	0.05 (<0.001)	0.03 (0.035)	0.01 (<0.001)	20.03	0.80
RV^2^	0.01 (0.043)	0.03 (<0.001)	0.03 (0.001)	0.01 (<0.001)	27.59	0.67
RV^3^	0.000 (0.628)	0.002 (<0.001)	0.000 (0.598)	0.001 (<0.001)	22.77	0.50
RV^4^	0.000 (0.286)	0.002 (<0.001)	0.000 (0.244)	0.001 (<0.001)	30.92	0.50
RV^5^	0 (0.945)	0.001 (<0.001)	0.009 (<0.001)	0.001 (<0.001)	55.85	0.00
RT^1^	4075.20 (0.871)	328543.55 (<0.001)	8363414.91 (<0.001)	127486.04 (<0.001)	19.43	0.61
RT^2^	1.24 (0.761)	23.51 (<0.001)	453.89 (<0.001)	17.45 (<0.001)	35.10	0.26
RT^3^	0.26 (0.395)	1.18 (<0.001)	0.06 (0.648)	0.92(<0.001)	78.67	0.22
RT^4^	0.11 (0.2)	0.56 (<0.001)	7.86 (<0.001)	0.71 (<0.001)	69.10	0.00
RT^5^	0.03 (0.001)	0.01 (<0.001)	0.02 (0.014)	0.01 (<0.001)	76.48	0.08

df, degree of freedom; PRL, primary root length; TRL, total root length; TSA, total root surface area; TRV, total root volume; RAD, root average diameter; TRT, total root tips; RF, root forks; RL^1-5^, RSA^1-5^, RV^1-5^, RT^1-5^ indicate average root length, root surface area, root volume and root tips in diameter between 0.0 and 0.5 mm, 0.5 and 1.0 mm, 1.0 and 1.5 mm, 1.5 and 2 mm and greater than 2.0 mm respectively; p-values were given in parentheses behind mean squares.

### Genetic correlations among tested traits

Pearson correlations coefficients among all the traits under two P regimes were analyzed and significant correlations (p<0.01 and p<0.001) were observed between pairs of traits (Table 3). Under OP condition, highly significant and positive correlation were obtained between TRL and TSA (r = 0.953), TSA and TRV (r = 0.953) followed by TRL and TRV (r = 0.855). The RF exhibited a highly significant correlation with TRL, TSA and TRV whereas under LP condition, a highly significant and positive correlation was observed for TRL and TSA (r = 0.951) followed by TSA and TRV (r = 0.929). Under both OP and LP conditions, TRV showed a highly significant and positive correlation with all other tested traits. The PRL and TRL showed significant and positive correlations with all other tested traits except RAD under both P regimes. RAD showed a significant negative correlation with TRT and RF under low P condition. The results showed that the relationship between PRL and TRL, PRL and TSA and TRV and TRT were the same under both P conditions, but RAD and TRT and RAD and RF were much smaller in OP than LP condition. The relationship between PRL and RAD, and RAD and TSA were also very different between OP and LP conditions.

**Table 3:** Pearson correlations among tested traits under optimumand low phosphorus conditions (sample size = 153).

OP	PRL	TRL	TSA	TRV	RAD	TRT	RF
PRL	1						
TRL	0.417***	1					
TSA	0.398***	0.953***	1				
TRV	0.350***	0.855***	0.953***	1			
RAD	0.058	0.149	0.379***	0.576***	1		
TRT	0.398***	0.685***	0.599***	0.496***	-0.089	1	
RF	0.249**	0.824***	0.807***	0.717***	0.097	0.526	1
**LP**							
PRL	1						
TRL	0.415***	1					
TSA	0.399***	0.951***	1				
TRV	0.335***	0.819***	0.929***	1			
RAD	-0.102	-0.146	0.121	0.397***	1		
TRT	0.329***	0.708***	0.611***	0.492***	-0.288**	1	
RF	0.262**	0.805***	0.723***	0.560***	-0.284**	0.577***	1

** and *** significant at p<0.01 and p<0.001.

OP, optimum phosphorus; LP, low phosphorus; PRL, primary root length; TRL, total root length; TSA, total root surface area; TRV, total root volume; RAD, root average diameter; TRT, total root tips; RF, root forks; ** and *** significant at p<0.01 and p<0.001 respectively.

### Comparison of root traits in different diameter classes under two phosphorus regimes

**Fig 2:**
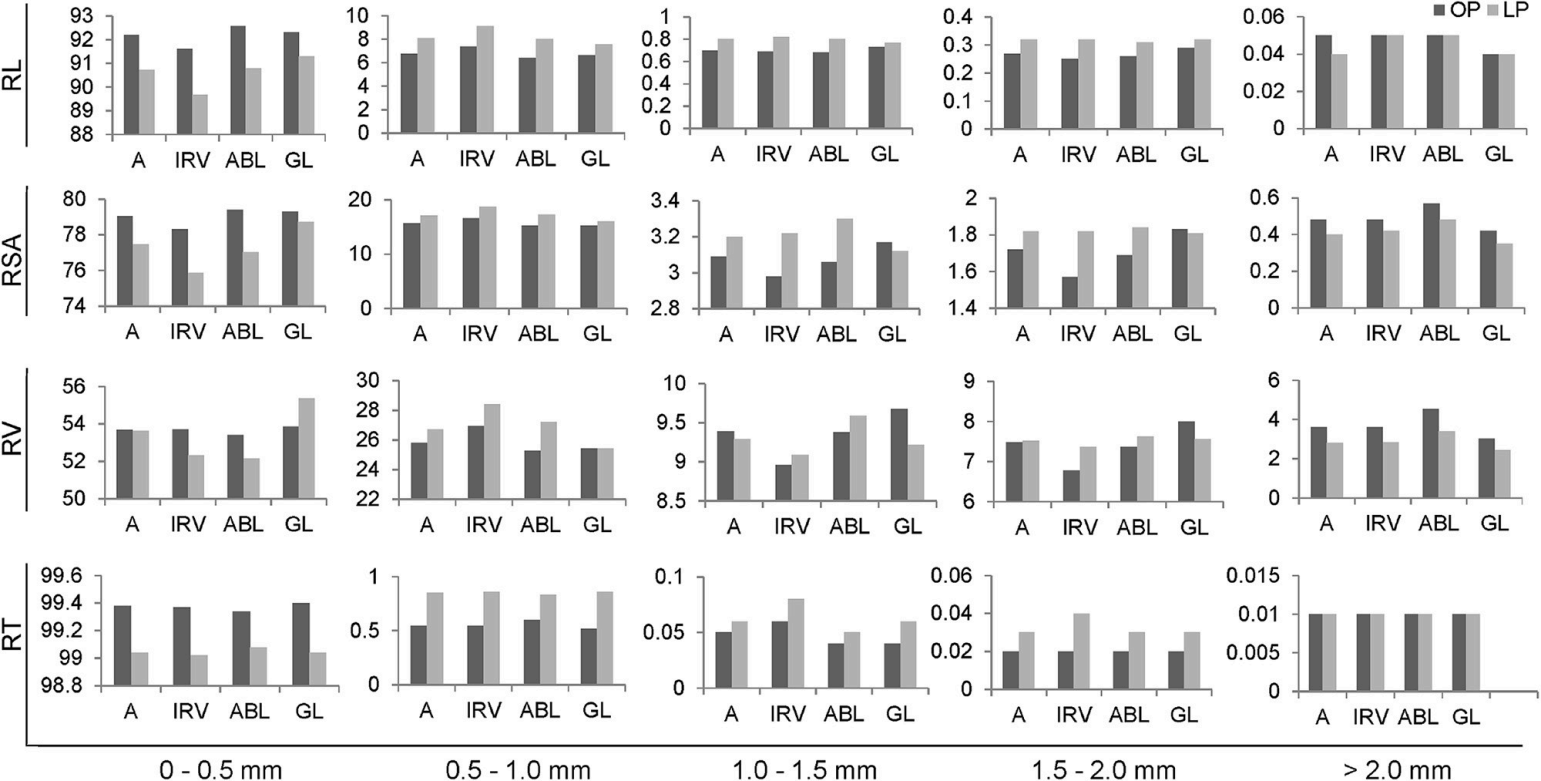
Percentage of root traits across five diameter classes assessed under two phosphorus regimes. RL, root length; RSA, root surface area; RV, root volume; RT, root tips; OP, optimum phosphorus condition; LP, low phosphorus condition; A, all 153 mungbean genotypes; IRV, Indian released varieties; ABL, advanced breeding lines; GL, germplasm lines.

Root traits TRL, TSA, TRV and TRT were classified into different classes based on root diameter intervals i.e. 0–0.5 mm, 0.5–1.0 mm, 1.0–1.5 mm, 1.5–2.0 mm and >2.0 mm and named them as RL^1-5^, RSA^1-5^, RV^1-5^ and RT^1-5^. This was done to compare the fine root distribution across different diameter intervals under both P conditions. The proportion of roots in each diameter class was calculated as a percentage of the total for each across different genotype groups under two P regimes (Table 4). The higher percentage distribution for studied root traits (RL, RSA, RV and RT) was recorded in 0–0.5 mm diameter class as compared to other diameter classes across different genotype groups under two P regimes. For root diameter classes 0.5–1.0 mm, 1.0–1.5 mm and 1.5–2.0 mm RL, RSA and RT percentage were higher in LP in comparison to OP condition (Fig 2). For diameter class in 0–0.5 mm and >2.0 mm, RL, RSA and RV recorded a higher percentage in OP condition as compared to LP condition.

**Table 4:** Percentage distribution of root traits across five root diameter classes under optimum and low phosphorus conditions in different groups.

Traits	Treatment	Root diameter class (mm)																			
		0–0.5				0.5–1.0				1.0–1.5				1.5–2.0				>2.0			
		A	IRV	ABL	GL	A	IRV	ABL	GL	A	IRV	ABL	GL	A	IRV	ABL	GL	A	IRV	ABL	GL
RL (%)	OP	92.20	91.61	92.58	92.32	6.78	7.39	6.43	6.62	0.70	0.69	0.68	0.73	0.27	0.25	0.26	0.29	0.05	0.05	0.05	0.04
	LP	90.73	89.68	90.80	91.30	8.11	9.13	8.03	7.57	0.80	0.82	0.80	0.77	0.32	0.32	0.31	0.32	0.04	0.05	0.05	0.04
RSA (%)	OP	79.06	78.32	79.41	79.30	15.66	16.64	15.27	15.28	3.09	2.98	3.06	3.17	1.72	1.57	1.69	1.83	0.48	0.48	0.57	0.42
	LP	77.48	75.85	77.04	78.73	17.10	18.70	17.34	16.00	3.20	3.22	3.30	3.12	1.82	1.82	1.84	1.81	0.40	0.42	0.48	0.35
RV (%)	OP	53.69	53.72	53.41	53.85	25.82	26.94	25.29	25.43	9.39	8.96	9.38	9.68	7.48	6.78	7.37	8.00	3.62	3.60	4.55	3.04
	LP	53.63	52.32	52.15	55.37	26.74	28.40	27.21	25.42	9.29	9.09	9.59	9.22	7.53	7.37	7.63	7.56	2.82	2.83	3.41	2.43
RT (%)	OP	99.38	99.37	99.34	99.40	0.55	0.55	0.60	0.52	0.05	0.06	0.04	0.04	0.02	0.02	0.02	0.02	0.01	0.01	0.01	0.01
	LP	99.04	99.02	99.08	99.04	0.85	0.86	0.83	0.86	0.06	0.08	0.05	0.06	0.03	0.04	0.03	0.03	0.01	0.01	0.01	0.01

OP, optimum phosphorus; LP, low phosphorus; A, all 153 mungbean genotypes: IRV, indianreleased varieties: ABL, advanced breeding lines: GL, germplasm lines; RL, root length; RSA, root surface area; RV, root volume; RT, root tips.

### Diversity pattern with respect to different groups

A comparison of the root morphology of the different groups showed clear variation for all studied root traits. The mungbean genotypes in the IRV, ABL and GL groups were classified into three categories, namely low, medium and high performance groups based on mean and standard deviation of each trait under both OP and LP conditions (Table 5). This classification mainly shows the frequency distribution of genotypes for studied root traits under both OP and LP conditions. For all studied traits, a larger number of genotypes were classified into the medium group. A critical review of performance under different P conditions revealed that among 153 genotypes studied, the greater number of genotypes were grouped in the high group in LP condition for PRL, TRL, TRV, RAD and TRT. Under LP condition, 8 (20%), 7 (16%) and 10 (15%) genotypes from IRV, ABL and GL groups showed larger TRL. For all traits except TSA, the GL group had a lower proportion of genotypes with high performance ($\geq \bar{x}+\text{SD}$) than either the IRV or ABL groups under two P regimes. Except for the trait TRL, ABL group had a lower proportion of genotypes with high performance (($\geq \bar{x}+\text{SD}$) than the IRV group under two P regimes. This indicates that more genotypes with P responsive root traits are present in the order IRV>ABL>GL groups under the two P regimes.

**Table 5:** The Shannon-Weaver diversity index (H’) and performance categories under two different phosphorus conditions.

Traits	Treatment	All 153 mungbean genotypes				Indian Released varieties				Advanced breeding lines				Germplasm lines			
		Low	Medium	High	H’	Low	Medium	High	H’	Low	Medium	High	H’	Low	Medium	High	H’
PRL	OP	25	102	26	0.87	5	28	8	0.85	8	30	6	0.84	14	44	10	0.89
	LP	22	103	28	0.83	4	29	8	0.79	5	29	10	0.86	11	48	9	0.81
TRL	OP	21	112	20	0.77	5	29	7	0.80	6	33	5	0.73	11	50	7	0.75
	LP	23	106	24	0.86	5	28	8	0.84	4	33	7	0.73	11	47	10	0.83
TSA	OP	22	109	22	0.80	6	30	5	0.77	5	33	6	0.74	9	49	10	0.79
	LP	21	110	22	0.79	5	29	7	0.80	4	35	5	0.65	13	46	9	0.85
TRV	OP	17	114	22	0.74	5	31	5	0.73	4	35	5	0.65	9	48	11	0.81
	LP	21	107	25	0.82	6	27	8	0.87	3	35	6	0.64	11	48	9	0.81
RAD	OP	17	113	23	0.75	9	25	7	0.94	4	35	5	0.65	12	45	11	0.87
	LP	28	101	24	0.88	5	29	7	0.80	6	29	9	0.87	10	52	6	0.70
TRT	OP	18	118	17	0.69	3	33	5	0.62	7	29	8	0.88	8	51	9	0.73
	LP	25	105	23	0.84	8	25	8	0.94	6	34	4	0.69	11	47	10	0.83
RF	OP	18	110	25	0.78	5	27	9	0.86	7	32	5	0.77	6	51	11	0.72
	LP	22	109	22	0.79	5	29	7	0.80	8	29	7	0.88	9	50	9	0.76

OP, optimum phosphorus; LP, low phosphorus; PRL, primary root length; TRL, total root length; TSA, total root surface area; TRV, total root volume; RAD, root average diameter; TRT, total root tips; RF, root forks.

The Shannon-Weaver diversity index (H’) was calculated to study the diversity among the tested traits in different genotypic groups (Table 5). The H’ values varied for traits PRL, TRL, TSA, TRV, RAD, TRT and RF with an average of 0.80 in mungbean genotypes. Among the studied groups H’ was maximum for Indian Released Varieties. Under OP condition, PRL and TSA exhibited higher H’ value in studied mungbean genotypes whereas in LP condition TRL, TRV, RAD, TRT and RF revealed higher H’ value. Under both Pregimes, RAD and PRL showed a relatively higher level of variation while TRV and RF were less variable across different genotypic groups. All root traits except PRL and TSA showed higher H’ values indicate higher diversity under the LP condition than the OP condition. For three traits, TRL, RAD and RF under OP condition and three traits, TRL, TRV and TRT under LP condition showed higher diversity in the IRV group than ABL and GL group.

### Principal component analysis

**Fig 3:**
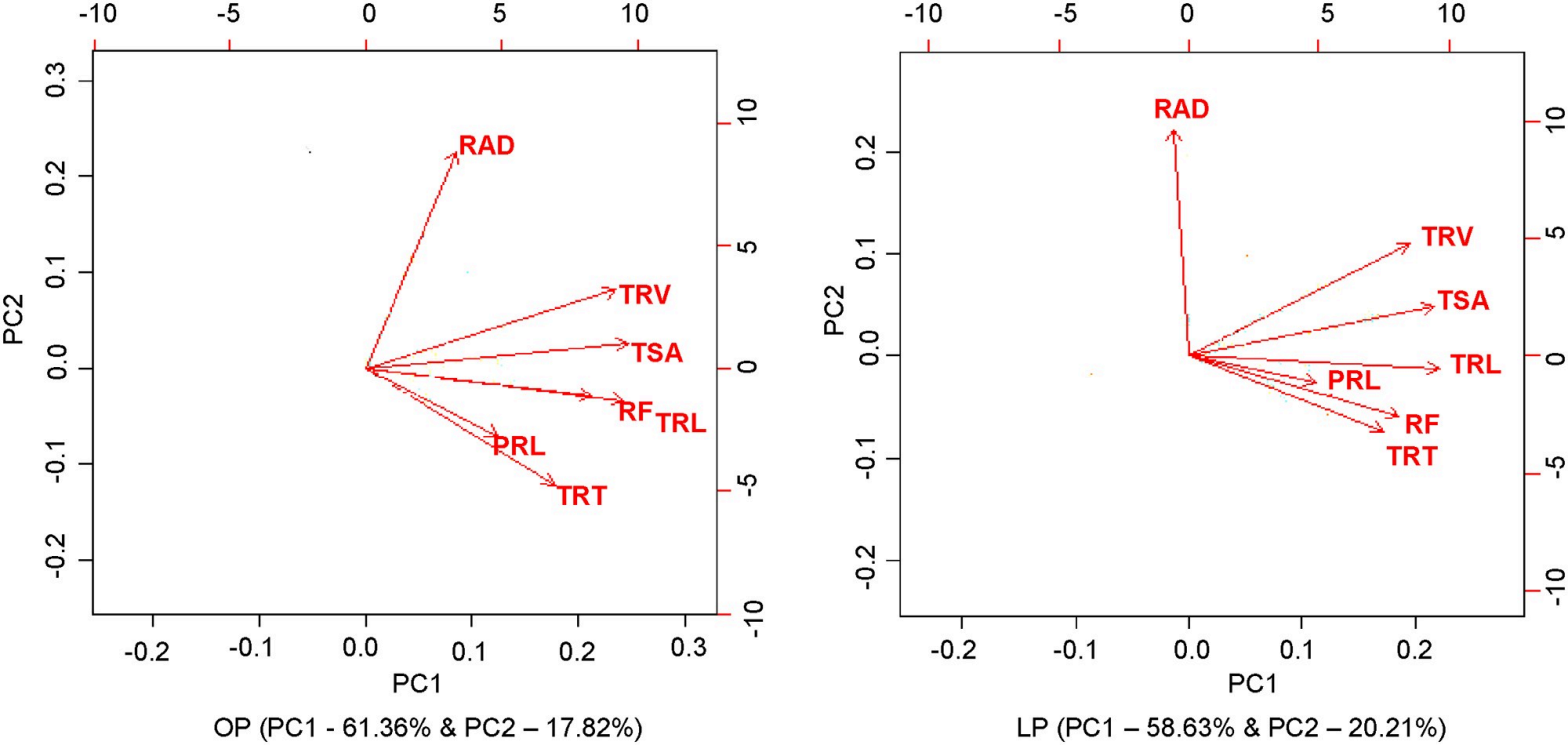
Biplots of first two principal components (PC) showing variation among seven root traits under optimum P and Low P condition. PRL, primary root length; TRL, total root length, TSA, total root surface area; RAD, root average diameter; TRV, total root volume; TRT, total root tips; RF, root forks; OP, optimum phosphorus condition; LP, low phosphorus condition.

The principal component analysis was carried out to know the most contributing traits under two P regimes. The first two principal components (PCs) explained 79.19% and 78.84% of the total variation among the tested mungbean genotypes under OP and LP conditions (Table 6 and Fig 3). The first principal component explained the 61% and 59% of total variation under OP and LP condition, revealed that TRL and TSA, and their highly correlated traits TRV and RF are the most important contributing traits. The most important contributing trait in the second principal component is RAD, which contributed nearly 20% of the total variation.

**Table 6:** Principle component analysis of seven traits under two phosphorus conditions.

Characters	OP		LP	
	PC1	PC2	PC1	PC2
PRL	0.24	-0.25	0.25	-0.09
TRL	0.46	-0.12	0.48	-0.05
TSA	0.47	0.09	0.47	0.18
TRV	0.45	0.29	0.42	0.41
RAD	0.16	0.80	-0.03	0.82
TRT	0.34	-0.43	0.37	-0.27
RF	0.41	-0.10	0.40	-0.22
EigenValues	4.29	1.25	4.10	1.41
% Variance	0.61	0.18	0.59	0.20
Cumulative % Variance	0.61	0.79	0.59	0.79
Most contributing traits	TSA, TRL, TRV	RAD	TRL, TSA, TRV	RAD

OP, optimum phosphorus; LP, low phosphorus; PC1, principal component 1; PC2, principal component 2; PRL, primary root length; TRL, total root length; TSA, total root surface area; TRV, total root volume; RAD, root average diameter; TRT, total root tips; RF, root forks.

### Comprehensive phosphorus efficiency measurement

**Fig 4:**
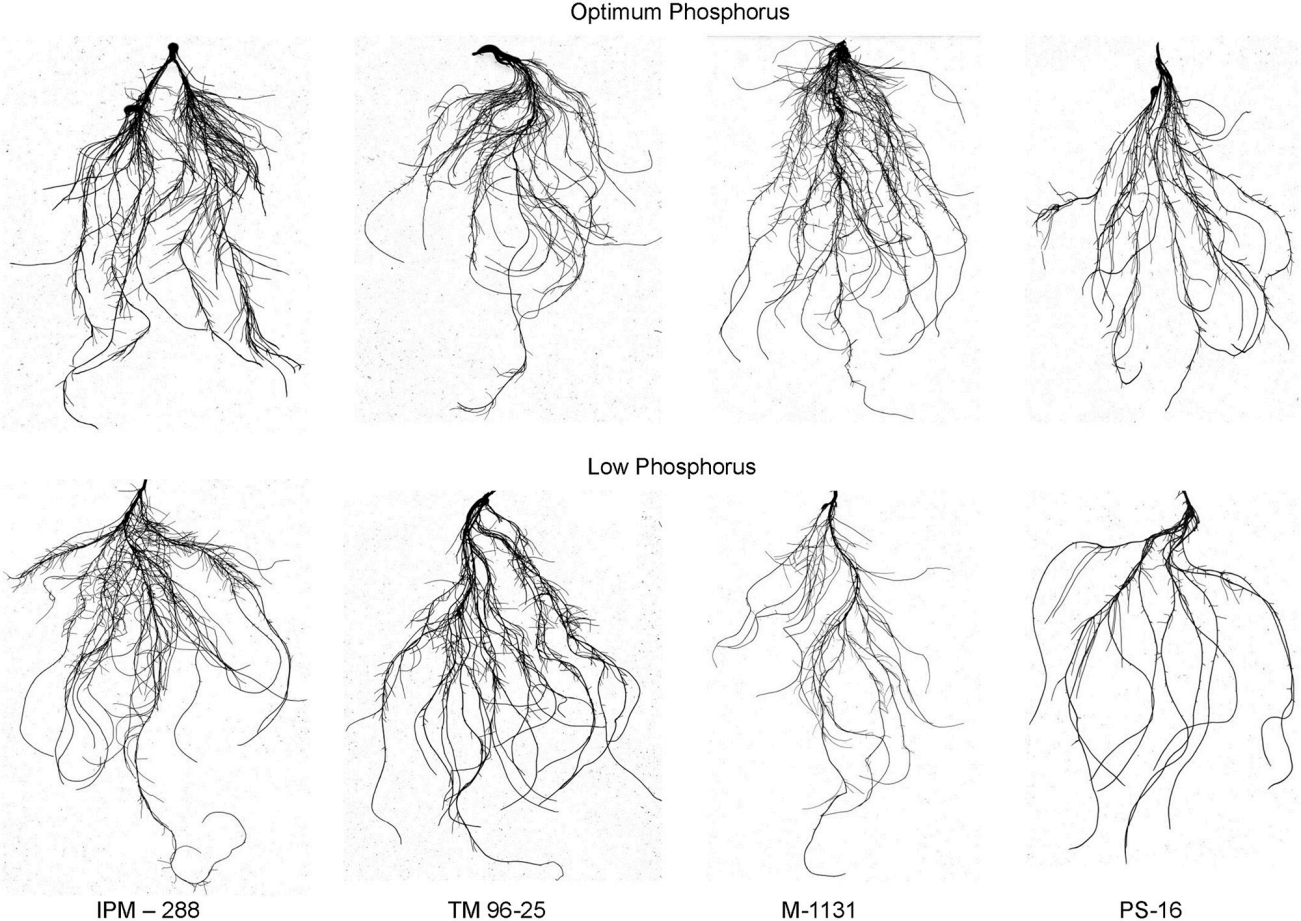
Variation in root system architecture in four contrasting mungbean genotypes grown under optimum and low phosphorus conditions.

**Fig 5:**
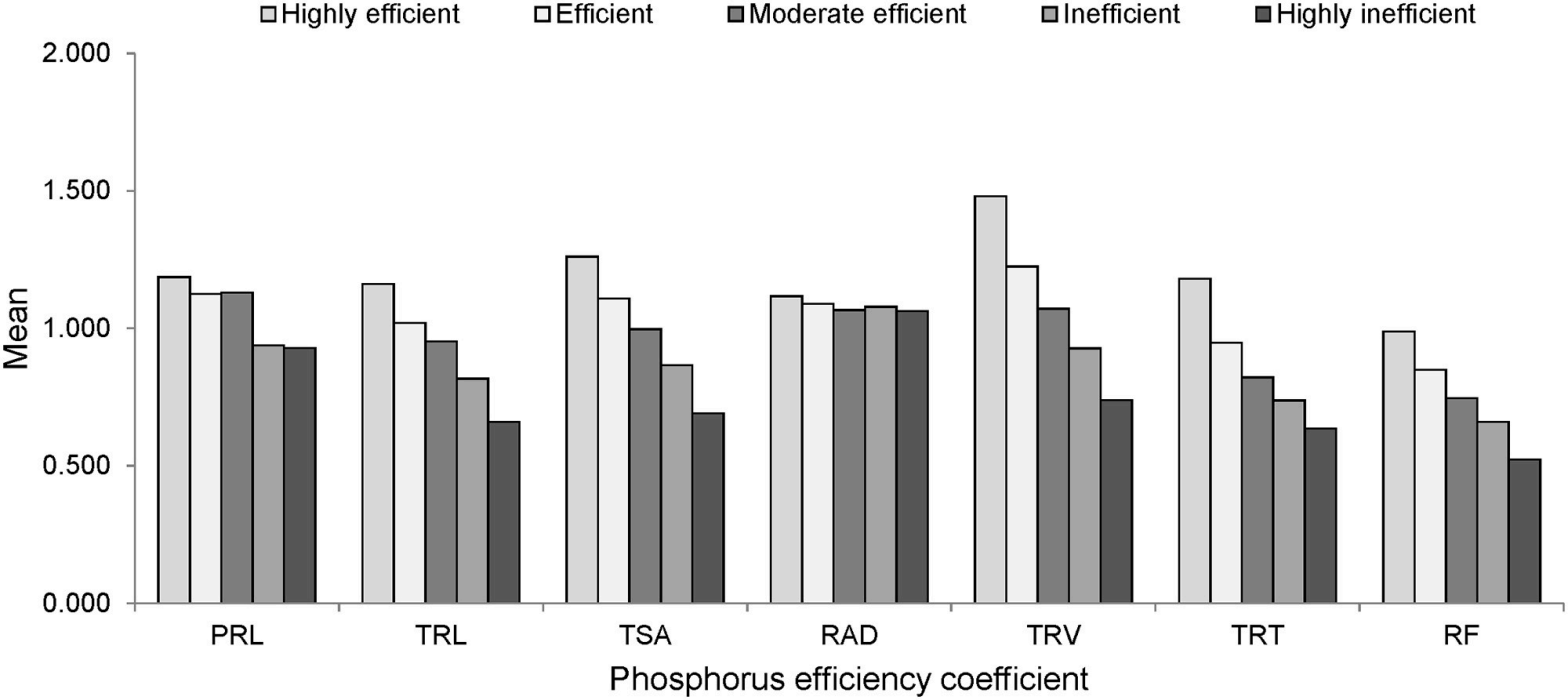
The mean values of phosphorus efficiency coefficient for seven traits in five groups classified for phosphorus efficiency. Groups 1, 2, 3, 4 and 5 represent mungbean genotypes identified with highly efficiency, efficiency, moderate efficiency, inefficiency and highly inefficiency. N = 21, 25, 48, 41 and 18 for groups 1, 2, 3, 4 and 5 respectively. PRL, primary root length; TRL, total root length; TSA, total root surface area; RAD, root average diameter; TRV, total root volume; TRT, total root tips; RF, root forks.

By using subordination function value analysis, the CPEM value, as a comprehensive synthetic index was derived to study the efficiency of root morphology among mungbean genotypes under P deficiency (S2 Table). Based on CPEM values, all mungbean genotypes were classified into five groups. Group 1 includes 21 genotypes showed high P efficiency with CPEM values greater than 0.9. Group 2 with 25 genotypes showed P efficiency with CPEM values between 0.7 and 0.9. Group 3 with 48 genotypes showed moderate efficiency with CPEM values between 0.5 and 0.7. Group 4 with 41 genotypes showed inefficiency with CPEM values between 0.3 and 0.5 while group 5 including 18 genotypes were highly inefficiency with CPEM values less than 0.3. The genotype which comes under respective groups is listed in S2 Table. Based on CPEM values, variation in root architectural traits of contrasting genotypes is presented in Table 7 and Fig 4. Genotype IPM-288 with the highest CPEM value recorded higher values for TRL, TSA, TRV and TRT under LP condition compared to OP condition. Genotypes with low CPEM values, M1131 and PS-16 were found to be poor performers for TRL, TSA and TRV under LP condition compared to OP condition. The mean values of the P efficiency coefficient for each trait in five groups with different levels of P efficiency are shown in Fig 5. The mean values of P efficiency coefficient for all root traits were highest in group 1, moderate in group 2, 3 and 4, and lowest in group 5 except for RAD. This result indicates that P efficient mungbean genotypes with higher CPEM values also had higher P efficiency coefficients.

**Table 7:** Variation in root architectural traits of ten contrasting mungbean genotypes under optimum and low phosphorus conditions.

Genotypes	CP EM	Root traits													
		PRL		TRL		TSA		TRV		RAD		TRT		RF	
		OP	LP	OP	LP	OP	LP	OP	LP	OP	LP	OP	LP	OP	LP
Top five genotypes															
IPM-288	1.77	25.00	32.67	839.70	1198.07	82.17	129.37	0.62	1.11	0.30	0.35	618.70	1067.33	2669.00	2301.33
TM 96–25	1.55	32.00	31.00	525.00	740.40	65.87	84.27	0.44	0.76	0.32	0.36	474.67	849.33	1517.33	1566.67
TM 96–2	1.48	44.33	41.00	777.53	932.53	70.67	100.20	0.51	0.86	0.29	0.34	728.33	1138.67	1817.33	2028.67
M 1477	1.37	28.83	32.33	631.75	740.74	60.34	78.67	0.44	0.72	0.30	0.35	594.00	653.33	1252.33	1644.33
PUSA 1342	1.27	29.33	44.00	734.35	928.65	73.73	96.42	0.52	0.80	0.31	0.33	898.33	742.00	1765.00	2017.33
Bottom five genotypes															
M 1131	0.18	38.00	33.00	843.25	509.77	85.62	49.83	0.61	0.37	0.31	0.31	900.67	703.67	2402.00	1388.33
PS—16	0.19	26.67	23.00	591.66	277.07	67.35	35.60	0.61	0.36	0.37	0.41	697.33	233.67	1107.33	448.67
PUSA VISHAL	0.19	38.00	32.33	1449.93	726.73	175.81	88.14	1.68	0.94	0.37	0.41	1222.00	475.00	3755.67	1204.00
M 831	0.22	41.00	32.17	945.84	662.59	95.35	63.94	0.65	0.49	0.30	0.31	1209.33	785.67	3234.00	1556.00
IC 325828	0.23	41.00	36.67	1061.36	709.37	113.83	77.45	0.95	0.67	0.35	0.35	960.67	793.00	3559.33	1502.33

Contrasting genotypes identified using Comprehensive phosphorus efficiency measurement value. PRL, primary root length; TRL, total root length; TSA, total root surface area; TRV, total root volume; TRT, total root tips; RAD, root average diameter; RF, root forks; OP, optimum phosphorus; LP, low phosphorus.

## Discussion

Characterization of mungbean genotypes for stress tolerance traits and screening for P efficient genotypes are indispensable for the success of the breeding programme. Conventionally, a higher root to shoot ratio has been considered as an index for P efficiency due to the increase in root biomass and large deep root system able to extract more nutrients [58, 59]. Total root length represents the sum of primary, seminal, crown, basal and lateral roots. The various components of the root system have also been selected as important traits for the screening of genotypes under P deficiency. In this study, we examined the influence of low P on root morphology of 153 mungbean genotypes and investigated the various root traits including PRL, TRL, TSA, TRV, TRT and RF. We found significant variation, medium to high heritability, approximately normal distribution and significant correlations for these root traits. Low coefficient of variation and high heritability of these traits indicates the genetic stability of the traits in the genotypes. The observed results were in agreement with reports of previous researchers [60–62]. The widely used indicator, RAD, was highly heritable suggesting that it is a reliable parameter for P efficiency. Frano *et al* [63] reported that root traits especially root diameter increases (hypertrophy) in response to abiotic stress conditions. The root diameter mainly controls the root length and surface area and results in bigger root dry weight [64].

Although the genetic variation of root system varies from plant to plant, the presence of very fine roots (<0.5 mm diameter) and fine roots (0.5 to 2.0 mm) determines the most percentage of root traits, is important for nutrient and water uptake [48, 65 and 66]. In this study, we identified the high percentage of fine roots in diameter class from 0.5 to 2.0 mm in LP compared to OP condition, while the percentage of very fine roots with <0.5 mm diameter was more in OP condition. This indicates the effect of P availability on the percentage of fine root distribution at different diameter classes in studied mungbean genotypes. Under low P, plants may increase the development of root cortical aerenchyma which enables the plant to maintain greater root diameter but reduce overall total root cost and root respiration [67, 68]. Greater fine root production results in increasing the overall adsorption area as an adaptive mechanism of stress tolerance [69].

The principal component analysis showed that TRL, TSA, TRV and RAD were responsible for most of the phenotypic variation at the seedling stage in the tested mungbean genotypes. TRL was significantly and positively correlated with TSA, TRV, TRT and RF under both OP and LP conditions. In combination with principal component analysis, we identified that TRL, TSA and TRV were sufficient to explain most of the variation and these were proved to be ideal traits for P efficiency screening at the seedling stage. Under the LP condition, RAD was significantly and negatively correlated with TRT and RF. This indicates that RAD is a key trait to differentiate P availability among the tested root traits. Moreover, these traits showed high P efficiency coefficient values in P efficient mungbean genotypes. This result is in agreement with previous reports. Pandey *et al*. [32] reported significantly higher root surface area and root volume in P efficient mungbean genotype under P stress. Root surface area has been found to be in close association with the nutrient absorption rate [70, 71]. Vigorous root growth with high root length and surface area ensures the efficient absorption of macro and micronutrients at the early growth stage of the plant [72]. Furthermore, root architectural traits mainly total root length and root number were significantly and positively correlated with biomass and grain yield [73, 74]. Therefore, the vigorous root system of the plant not only supports good crop establishment but also ensures the plants’ survival under stressful conditions.

Diversity in root architecture enables us to improve nutrient and water use efficiency under stressful conditions. A combination of availability of diverse mungbean genotypes and stress tolerance ability will be key criteria for the success of crop improvement programme. Considering the mean performance and standard deviation of root traits as selection criteria, genotypes were categorized into high, medium and low performance groups [46, 51]. Furthermore, the Shannon–Weaver diversity index (H’) was calculated based on the categorization of genotypes to compare the phenotypic diversity among the traits. Among all traits, TRL and RAD (>0.8) showed a relatively high level of H’ under LP condition and PRL showed the relatively highest value of H’ (>0.8) under both P regimes. Independently of root length, greater diversity in diameter is due to the changes in the fine root distribution for each root diameter in response to the nutritional environment [75]. The high value of H’ indicates greater phenotypic diversity and balanced frequency distribution [76], while low H’ indicates an extremely unbalanced frequency distribution with a lack of diversity [77]. The Shannon-Weaver diversity index has been used previously to describe root traits diversity in rice [78], maize [79], wheat [80] and cowpea [81].

In this study, a comparison of root morphological traits across different genotypic groups indicated that the IRV group showed greater diversity for root traits than ABL and GL groups. The presence of high H’ values in the IRV group for TRL, TSA, TRV and TRT with medium to high heritability and number of genotypes in the high group in LP condition indicates that these genotypes used in the study are a rich source to improve the plant performance under LP. Results from broad-sense heritability, Shannon-weaver diversity index and principal component analysis facilitate the root architectural traits suitable for target genotype selection in cowpea [81]. Presence of more variable root architectural traits with high heritability suggests that genotypes are more P efficient in soybean under P limiting condition [82]. Among tested traits, RF was found to be less variable root trait conferring P efficiency in this study. In maize, RF was less variable due to less H’ value in all studied lines in response to water stress condition at the seedling stage [46]. Root traits, PRL and RAD were observed to be more variable across different genotypic groups under two P regimes. Further mean values of both these traits were significantly high in LP compared to OP condition and can be used as indicators of P deficiency. Plasticity of root traits including root length, root average diameter and percentage of lateral roots confers the improved plant performance under P stress condition [83]. Reduction of root diameter was reported under low P compared sufficient P condition in maize [62] and *Aegilops tauschii* [84]. Term ‘root etiolation’ has been suggested for the reduction in root diameter under P stress in common bean genotypes to increase soil exploration and to reduce metabolic cost [85]. In response to P deficiency growth of PRL was observed to be inhibited in *Arabidopsis* [86]. Whereas, enhanced induction of primary root was observed in rice [87]. Reduction in growth of primary root of P deficient plants correlates with inhibition of cell differentiation in primary root meristem and the reduction of cell proliferation in the root elongation part [88]. However, in present study, a comprehensive index CPEM has been estimated considering all the tested traits with irrespective of the nature of trait. For example, genotype with shorter or longer PRL, CPEM will consider ratio of trait value derived from LP and OP conditions. Further, CPEM is based on relative trait values i.e. PEC of traits and degree of membership between trait value and P efficiency i.e. MFVP [89]. In combination with correlation and principal component analysis, TRL with high H’ value and significant and positive correlation with TSA and TRV indicates that these traits are sufficient to explain the variation and could be used as selection criteria for P efficiency at the seedling stage. In maize, total root length and root dry weight were able to provide the most contribution to total phenotypic variation and sufficient to improve other root traits [46, 79]. This result provides valuable information to improve both agronomic traits as well as nutrient use efficiency traits in the mungbean breeding programme. In the 21^st^ century, due to environmental concerns and the high cost of inorganic fertilizers, nutrient efficient crop plants play an important role in improving crop yields compared to 20^th^ century [90].

Based on CPEM values, mungbean genotypes were classified as highly efficient, efficient, moderately efficient, inefficient and highly inefficient groups. Among these, IPM-288, TM 96–25, TM 96–2, M-1477, PUSA 1342 were identified as best five highly efficient genotypes whereas M-1131, PS-16, Pusa Vishal, M 831, IC 325828 were highly inefficient genotypes. Except for RAD, P efficiency coefficients for all traits were highest in group 1, intermediate in group 2, 3 and 4 while lowest in group 5. This type of classification is required for screening and selection of genotypes for desirable root traits under varied P conditions. Further, these genotypes with contrasting traits can be exploited in recombination breeding programme to develop P efficient cultivars [91, 92]. In this study, 21 highly efficient genotypes with a well developed root system were identified and these could be used in the mungbean breeding programme for further improvement of tolerance to abiotic stresses.

In conclusion, the veracity of the root system was maintained by growing the tested mungbean lines in hydroponic culture. The *in vitro* screening method of hydroponics proves to be the ideal method to screen large set genotypes with the least effect of environmental influence [93]. Previous reports examined the root traits using the simple hydroponic system based on the aerated nutrient solution with different levels of P with the replacement of solution at a fixed interval in maize [94], soybean [95], mungbean [32] and wheat [96]. In the present study, we identified a range of responses to P deficiency in mungbean genotypes for root system traits at the seedling stage. We found that TRL, TSA and TRV are the ideal selection criteria at the seedling stage for predicting the nutrient use efficiency in the field. Thus, the root system response to P deficiency can be studied without root damage in hydroponic culture by controlling access to water and nutrients. Further, the tested mungbean genotypes need to be evaluated at the adult stage under OP and LP conditions. In addition, the association of seedling stage root traits with adult stage traits needs to be further examined.

## Supporting information

S1 Fig Effect of increasing phosphorus concentration on (A) chlorophyll concentration and (B) total biomass in 21 days plants of mungbean genotype (PUSA-9072). (PDF)

S1 Table List of 153 genotypes of mungbean used in the study. (PDF)

S2 Table Comprehensive phosphorus efficiency measurement value of 153 mungbean lines used in the study. (PDF)
