# Supplementary material for: Genetic variation for root architectural traits in response to phosphorus deficiency in mungbean at the seedling stage
Source: PLoS One. 2020 Jun 11;15(6):e0221008. doi: 10.1371/journal.pone.0221008 (PMC7289352; doi:10.1371/journal.pone.0221008)

S1 Fig. Effect of increasing phosphorus concentration on (A) chlorophyll concentration and (B) total biomass in 21 days old plants of mungbean genotype (PUSA-9072)

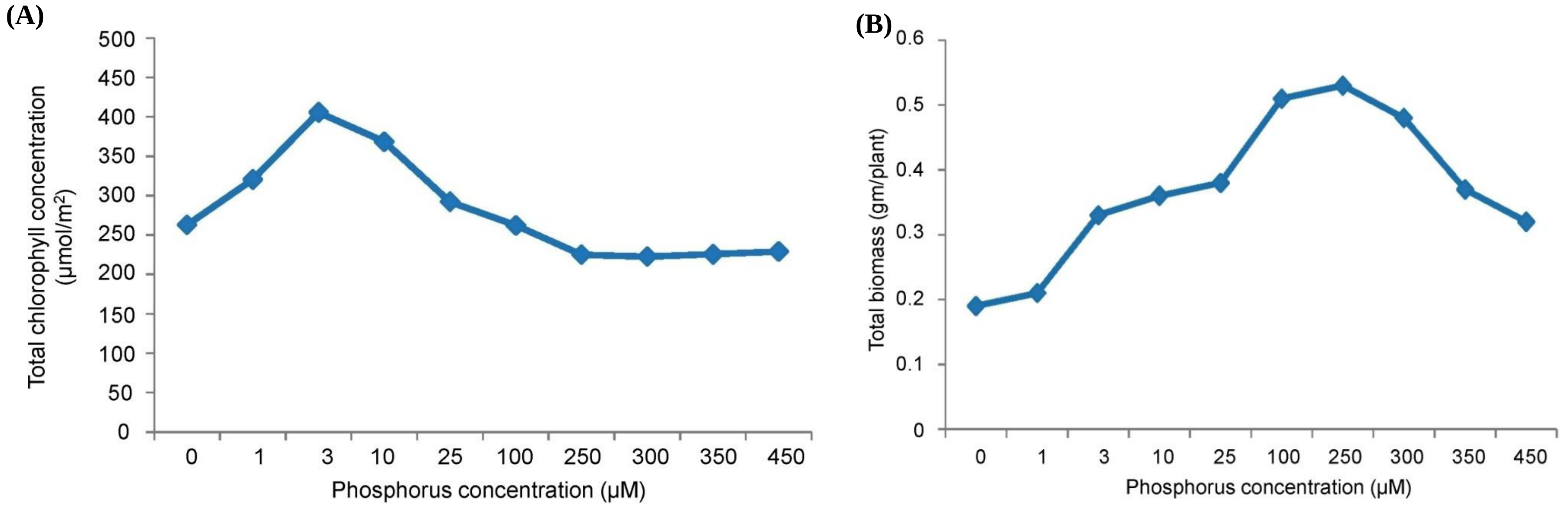

Supplement: S1 Fig — (PDF) [file pone.0221008.s001.pdf]
