# Supplementary material for: Genetic variation for root architectural traits in response to phosphorus deficiency in mungbean at the seedling stage
Source: PLoS One. 2020 Jun 11;15(6):e0221008. doi: 10.1371/journal.pone.0221008 (PMC7289352; doi:10.1371/journal.pone.0221008)
Supplement: S1 Table — (PDF) [file pone.0221008.s002.pdf]

S1 Table. List of 153 genotypes of mungbean used in the study.

S. No	Group	Genotypes
1	Indian Released Varieties (IRV)	ML-818, PDM-139, PS-16, Pusa Vishal, Pusa Ratna, MH 215, MH 96-1, MH 318, HUM 1, HUM 2, HUM 6, HUM 16, IPM-02-3, IPM-02-14, IPM-205-7, Ganga 8, TM 96-2, TM 96-25, AKM 9904, COGG 912, LGG 460, RMG 991, RMG 1087, MASH 114, Bhutan Lm 1, Bhutam Lm 2, Bhutan Lm 95, China Mung, Prakash Nephal, RMGP 1, IPM 410-3, RMG 1028, PUSA 0971, GANGA 1, NM 1, Muskan, Satya, MH 810, Basanti, Pusa Baisakhi, SML 668
2	Advanced Breeding lines (ABL)	Pusa 871, Pusa 1033, Pusa 1333, MH 565, IPM-02-15, IPM-02-17, IPM-02-30, IPM-288, IPM-205-4, IPM 406-1, IPM 409-4, KM 11-10, V 1138, V 3518, V 6183, KM 16-69, KM 16-75, KM 16-60, KM 16-81, KM 16-80, KM 16-76, KM 16-82, KM 16-58, KM 16-23, KM 2241, PUSA 1131, PUSA 1132, PUSA 1341, PUSA 1342, PUSA 1331, PUSA 1332, PUSA 1441, KM 7-134, KM 12-28, KM 11-40, IPM 02-19, TM 9725, M 1319B, MH 934, MH 1442, ML 1628, V 1109, V 1153, DMS 8
3	Germplasm lines (GL)	ML 512, ML 1299, ML 1464, ML 2037, EC 3988891, EC 520024, EC 520026, EC 520041, EC 550851, PLM 167, PLM 271, V 04-04, M 512, M 145, M 703, M 678, M 204, M 684, M 765, M 1168, M 1477, M 981, M 1503, M 875, M 201, M 1378, M 723, M 1358, M 700, M 906, M 831, M 1370, M 958, M 880, M 1131, M 1255, M 42, M 460, M 1156, M 1447, M 260, M 289, M 499, M 1032, M 837, M 313, M 1053, M 1372, M 1485, ML 1451, M 422, OLRM 4, OLRM 24, EC 520029, IC 546488, IC 436763, IC 28083, IC 436637, IC 325828, IC 282094, IC 282096, IC 546476, M 565, M 1400, M 1421, M 1493, YM 1, YM 2
