# Supplementary material for: Genetic variation for root architectural traits in response to phosphorus deficiency in mungbean at the seedling stage
Source: PLoS One. 2020 Jun 11;15(6):e0221008. doi: 10.1371/journal.pone.0221008 (PMC7289352; doi:10.1371/journal.pone.0221008)
Supplement: S2 Table — (PDF) [file pone.0221008.s003.pdf]

S2 Table. Comprehensive phosphorus efficiency measurement value of 153 mungbean lines used in the study

S. No	Class	Genotypes and P value
1	Highly efficient	IPM-288 (1.77), TM 96-25 (1.55), TM 96-2 (1.48), M 1477 (1.37), PUSA 1342 (1.28), V 6183 (1.27), M 703 (1.26), M 42 (1.25), PUSA 1033 (1.17), M 1168 (1.16), M 906 (1.16), MH 565 (1.14), M 1032 (1.13), M 765 (1.05), MASH 114 (1.03), V 1153 (0.99), HUM 6 (0.97), KM 16-60 (0.96), GANGA 8 (0.95), IC 436637 (0.95), M 422 (0.94)
2	Efficient	HUM 16 (0.93), ML 1451 (0.89), LGG 460 (0.88), OLRM 24 (0.88), EC 520041 (0.85), MH 934 (0.85), BASANTI (0.85), M 1503 (0.85), M 460 (0.85), IC 282096 (0.83), RMG 1087 (0.82), MH 810 (0.81), KM 16-75 (0.8), NM 1 (0.79), M 201 (0.79), TM 9725 (0.78), MUSKAN (0.78), M 1493 (0.78), Pusa 871 (0.77), M 678 (0.77), M 1255 (0.77), V 1138 (0.76), PUSA 1341 (0.76), M 499 (0.76), M 837 (0.76)
3	Moderate efficient	M 1370 (0.75), V 1109 (0.75), ML 2037 (0.74), M 723 (0.74), M 1372 (0.74), M 1485 (0.74), IC 546488 (0.74), PUSA 1331 (0.73), M 1447 (0.73), M 958 (0.72), COGG 912 (0.71), V 3518 (0.71), KM 16-58 (0.71), M 289 (0.7), M 565 (0.7), EC 3988891 (0.68), RMG 1028 (0.68), M 204 (0.67), KM 16-82 (0.66), M 1053 (0.66), KM 16-81 (0.65), ML 1464 (0.64), PUSA 0971 (0.64), GANGA 1 (0.64), EC 550851 (0.63), IC 28083 (0.63), V 04-04 (0.62), YM 2 (0.62), KM 16-80 (0.61), HUM 1 (0.61), YM 1 (0.61), EC 520024 (0.6), PLM 167 (0.6), KM 16-69 (0.58), IC 436763 (0.58), M 1421 (0.58), IPM-02-3 (0.57), Prakash Nephal (0.57), KM 16-23 (0.57), Pusa Baisakhi (0.57), M 260 (0.57), IC 282094 (0.57), ML 512 (0.56), PDM 139 (0.56), Bhutan Lm 95 (0.56), China Mung (0.56), ML 1628 (0.56), HUM 2 (0.51)
4	Inefficient	IPM-02-15 (0.54), KM 11-10 (0.53), M 875 (0.53), IPM 02-19 (0.51), M 684 (0.51), SATYA (0.5), M 1156 (0.5), IPM-02-14 (0.49), IPM 406-1 (0.49), IPM 409-4 (0.49), PUSA 1131 (0.49), M 313 (0.49), MH 215 (0.48), ML 1299 (0.48), KM 2241 (0.48), PUSA 1332 (0.48), IPM-205-4 (0.47), M 880 (0.47), IC 546476 (0.47), Pusa Ratna (0.46), AKM 9904 (0.46), RMGP 1 (0.46), PUSA 1132 (0.46), ML 818 (0.46), M 1400 (0.46), KM 16-76 (0.45), KM 12-28 (0.44), M 1358 (0.44), Bhutan Lm 1 (0.43), M 1378 (0.43), DMS 8 (0.43), Bhutan Lm 2 (0.42), SML 668 (0.42), OLRM 4 (0.42), MH 318 (0.41), M 145 (0.41), PUSA 1333 (0.39), MH 96-1 (0.39), IPM 410-3 (0.38), PUSA 1441 (0.38), M 981 (0.38)
5	Highly inefficient	KM 7-134 (0.37), IPM-02-30 (0.36), EC 520026 (0.36), M 1319B (0.36), EC 520029 (0.36), RMG 991 (0.35), M 512 (0.35), M 700 (0.35), IPM-205-7 (0.32), KM 11-40 (0.31), IPM-02-17 (0.29), MH 1442 (0.29), PLM 271 (0.27), IC 325828 (0.23), M 831 (0.22), Pusa Vishal (0.19), PS-16 (0.19), M 1131 (0.18)
